# Liking and wanting pleasant odors: different effects of repetitive exposure in men and women

**DOI:** 10.3389/fpsyg.2014.00526

**Published:** 2014-05-30

**Authors:** Chantal Triscoli, Ilona Croy, Håkan Olausson, Uta Sailer

**Affiliations:** ^1^Clinical Neuroscience and Rehabilitation, Institute of Neuroscience and Physiology, University of GothenburgGothenburg, Sweden; ^2^Department of Psychology, University of GothenburgGothenburg, Sweden

**Keywords:** wanting, liking, odor, pleasantness, gender, smells, odors

## Abstract

Odors can enrich the perception of our environment and are commonly used to attract people in marketing situations. However, the perception of an odor changes over repetitions. This study investigated whether repetitive exposition to olfactory stimuli leads to a change in the perceived pleasantness (“liking”) or in the wish to be further exposed to the same olfactory stimulus (“wanting”), and whether these two mechanisms show gender differences. Three different pleasant odors were each repeatedly presented for 40 times in random order with a mean inter-stimulus interval of 18 s. Eighteen participants rated both “liking” and “wanting” for each of the 120 olfactory stimuli. Wanting ratings decreased significantly over repetitions in women and men, with a steeper decrease for men during the initial trials before plateauing. In contrast, liking ratings decreased significantly over repetitions only in men, with a steeper decrease after the initial ratings, but not in women. Additionally, women scored higher in a questionnaire on reward responsiveness than men. We conclude that positive evaluation (liking) and the wish to experience more of the same (wanting) are different concepts even in the domain of olfaction. The persistence of perceived pleasantness in women may be due to the attribution of a greater subjective value to odors.

## Introduction

The sense of smell plays an important role in everyday life. As olfactory stimuli signal the presence of food and threat, amongst other things, they affect our behavior and subsequent actions (Gottfried et al., 2002). Odors can induce subjective feelings of pleasure and make us come back for more. It has been suggested that these two aspects, the experienced pleasantness of a stimulus and the motivation to obtain the stimulus, represent two separate and independent aspects (Berridge, 2009) that have to be differentiated from each other. For example, a strong urge to obtain a certain pleasant sensory stimulus may not necessarily mean that one subsequently enjoys its consumption. These two aspects have been termed “liking” and “wanting” (Berridge, 2009) and are thought to be mediated by different brain substrates. Indeed, pharmacological manipulations of these brain areas can alter “wanting” without affecting “liking” and vice versa (Berridge, 1996, 2009; Berridge et al., 2009). There is a rich body of literature on liking and wanting relating to food (Berridge, 1996, 2009; Berridge et al., 2009; Berridge and Robinson, 2011) and to the effects of drugs (Wise, 1980, 1996; Koob, 1992; Harriet, 1996; Spanagel and Weiss, 1999; Kelley and Berridge, 2002). However, a whole range of other stimuli can also cause positive hedonic experiences, such as odors (Gottfried and Wilson, 2011; Rolls, 2014), pleasant touch (Kida and Shinohara, 2013a,b; Rolls, 2014), social connection (Morelli et al., 2014), and music (Menon and Levitin, 2005; Montag et al., 2011; Salimpoor et al., 2013), to only name a few. It is as yet unknown whether the differentiation into wanting and liking also applies to these other types of stimuli, for example, odors.

The subjective value of odors may also be processed differently depending on gender. Although men and women have been reported to have similar sensory abilities when detecting and discriminating odors (Oberg et al., 2002), there are studies showing a female advantage for familiarity and recognition of odors (Brand and Millot, 2001), remembering (Klukty, 1990; Oberg et al., 2002) and identifying odors (Doty et al., 1985; Ferdenzi et al., 2013). Women were also found to more easily associate an odor with a term, pointing at better semantic abilities linked to odors (see also Ferdenzi et al., 2013). However, because of their enhanced association ability, it is likely that women's superiority in these olfactory tasks is due to cognitive rather than sensory factors.

A further aspect of olfaction in which men and women seem to differ is the impact of the sense of smell in everyday life. This is suggested by responses in a questionnaire evaluating the subjective importance of the sense of smell (Croy et al., 2010), in which women were found to attribute a higher importance to olfaction than men did. In the same way, a different study reported a higher interest in the sense of smell for women than for men (Seo et al., 2011). This larger interest or importance can be expected to lead to gender differences in pleasantness ratings.

Furthermore, wanting and liking may change with repeated exposure. The intensity and perception of odors has been found to change due to habituation and potential desensitization processes (Andersson et al., 2013). However, changes in the subjective value of an odor may be independent of habituation. For example, it has been reported that the perceived pleasantness (“liking”) of pleasant odors was maintained over repetitions, whereas the perceived unpleasantness of malodors decreased (Croy et al., 2013b). To prevent habituation, these authors used effect of habituation a long inter-stimulus interval of 22 s. Applying 96 olfactory stimuli in a row, they found no signs of habituation for pleasant odors as measured by intensity ratings and evoked potentials (Croy et al., 2013b).

The potential persistence of the experienced pleasantness of odors is relevant for marketing situations. Odors are not only ubiquitous in shops and restaurants, but are also used for shop design (Soars, 2009). The perception of diffused pleasant odors has been shown to contribute to a positive evaluation of a mall environment, and indirectly to a product's quality (Chebat and Michon, 2003). When pleasant odors were used, shoppers perceived the time spent in a store as shorter, and their overall perception of the environment, their purchase intention, and the likelihood to revisit the store were improved, irrespective of the nature and the intensity of the odor (Spangenberg et al., 1996). Another study demonstrated that lavender odor diffused in a restaurant, compared to a non-smell condition, increased the duration of stay for customers and the amount purchased (Gueguen and Petr, 2006). Finally, a study conducted in a real-life situation (a shopping mall), showed that odors positively influenced shoppers' perceptions, but only when the shop was neither crowded nor empty (Michon et al., 2005). Thus, if the perceived pleasantness of an odor would remain constant over repetitions, perfuming shops could be considered as a selling strategy to keep customers longer inside a shop.

The present study aimed to investigate whether “wanting” and “liking” of odors develop differently over repetitions. Furthermore, we expected that gender influences the wanting and liking of odors, because women have been reported to attribute greater importance to the sense of smell than men do.

## Methods

### Participants

In total, 18 subjects, aged between 20 and 36 years (*M* = 27, *SD* = 3.8) were recruited, 9 of them were men and 9 of them were women. The majority of the participants were students; some of them had already taken part in a previous, but unrelated experiment on touch perception. The participants were asked not to join the experiment if they were suffering from a cold, in order to avoid reduced olfactory performance. All the participants signed an informed consent form and received a compensation for participating in the study (200 SEK per hour).

The study was approved by the ethics committee of the University of Gothenburg.

### Materials

The odor stimuli were delivered in opaque glass bottles (50 ml capacity) containing odorant diluted in propylene glycol (Sigma Aldrich, Steinheim, Germany).

A first **pre-test** served to establish concentrations that were equal in subjective intensity. The pre-test and its results are described in the following paragraph.

The sample consisted of 18 students (16 females, 2 males), aged between 19 and 42 years (*M* = 28, *SD* = 6.91), none of which participated in the later experiment. Four different odors were presented at 3 different levels of concentration. These odors were ready-made perfume mixtures (Firmenich, Kerpen, Germany) smelling of flowers (diluted to 1.8, 5.5, 16.6%), aloe (diluted to 0.49, 0.96, 1.8%), vanilla (diluted to 0.5, 0.96, 1.8%), and coconut (diluted to 1.8, 5.5, 16.6%). Two further odors not relevant for the present study were presented in 4 different levels of concentration. Thus, 20 different stimuli resulted which were presented in random order. Pleasantness and intensity were rated on an 11-point scale, pleasantness: −5 (extremely unpleasant) to 5 (extremely pleasant); intensity: 0 (not intense at all) to 10 (extremely intense). The mean ratings for all participants were then submitted to two separate repeated-measures ANOVAs with the factors concentration (low, middle, high) and odor (flower, aloe, vanilla, coconut). Based on these results, those concentrations were selected that were found to differ in perceived pleasantness, but not in intensity (for mean rating values, see Table 1). This was the case for coconut (16.6%), vanilla (0.9%), aloe (0.49%), and flowers (5.5%). Pairwise comparisons with Bonferroni-corrections showed that vanilla was perceived as less pleasant than both flowers (*p* < 0.01) and aloe (*p* < 0.05). Aloe was also perceived as more pleasant than coconut (*p* < 0.05). For the subsequent experiment, we decided to use the odors coconut, aloe and flowers, since these three were on average clearly experienced as pleasant, as compared to vanilla.

**Table 1:** **Mean pleasantness ratings and standard deviations in parentheses for concentrations similar in perceived intensity, but differing in perceived pleasantness**.

	**Coconut (16.6%)**	**Vanilla (0.9%)**	**Aloe (0.49%)**	**Flowers (5.5%)**
Intensity	5.78 (1.99)	5.56 (1.76)	5.83 (2.01)	5.39 (3.15)
Pleasantness	1.33 (2.09)	−0.17 (2.15)	2.94 (1.11)	2.11 (1.68)

#### Experimental setting and procedure

Prior to the experiment, normal olfactory function was ascertained with the use of the “Sniffin' Sticks” odor identification test (Burghart Instruments, Wedel, Germany) (Kobal et al., 1996). The maximal score to obtain in this test is 16. In the present study, subjects were included when they had at least 10 correct answers. The probability of having 10 or more answers right by pure chance is 0.16%. All the subjects attained this criterion.

The participants were asked to sit on a comfortable chair and to make their ratings on an iPad (Apple Inc., Cupertino, USA), which was connected to a PC via iDisplay (SHAPE, Stuttgart, Germany). The participants wore headphones in order to be able to better concentrate on their sense of smell and not to be distracted. Subjects were instructed to smell the three odors flowers, aloe and coconut, in the concentrations established in the pre-test and grade their pleasantness on the iPad in front of them. The odors were contained in opaque glass bottles that were labeled X, Y, Z. Subjects were told to breathe deeply during the break between the different smells. Odor presentation was randomized within 40 triplets (each triplet consisting of aloe, flowers, and coconut). Thus, each odor was presented 40 times, resulting in 120 trials. The whole session lasted about 36 min.

**Figure 1:**
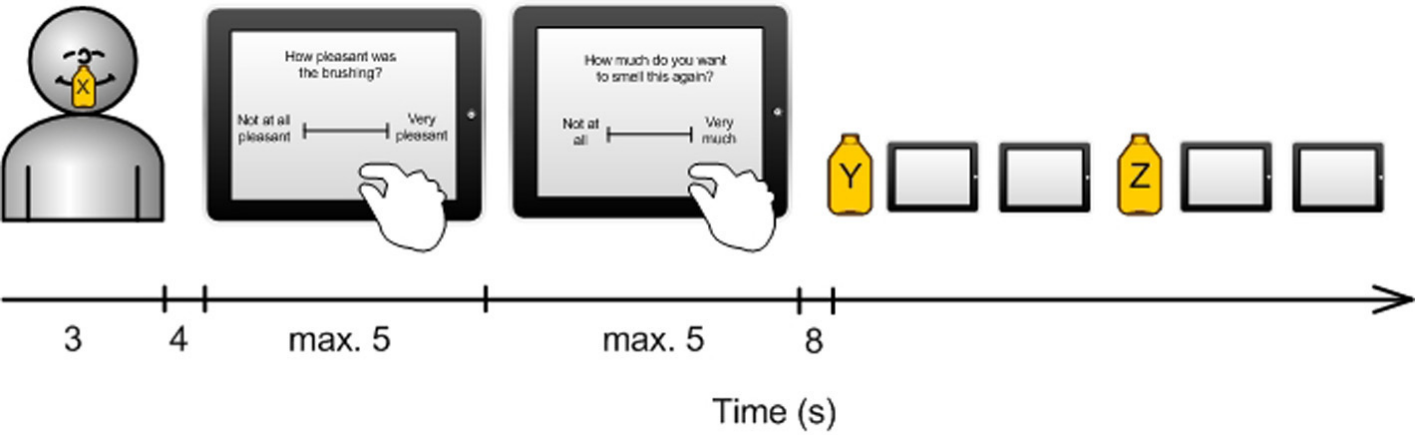
**Timeline of the experimental procedure**. One trial consisted of the presentation of one smell and 2 subsequent rating scales (for explanation, see text).

Odor presentation was guided by a computerized experimental protocol (programmed in MATLAB, Mathworks, Natick, MA) visible only to the experimenter. It showed a count-down to present the odor to the subject at the right time, the type of odor to administer (X, Y, Z) and the subsequent one. The average of the inter-stimulus interval between the presentations of each odor was 18.27 s, thus, 15 s plus the reaction times for liking and wanting. A count-down was shown on the screen. The experimenter sat next to the subject and opened the bottle indicated by the program 8 s after the count-down started. The odor was held directly under the subject's nostrils for 3 s. During the remaining 4 s the subjects waited for the rating scale to appear. We chose a duration of 4 s so that the subjects had time to think about which rating to give. The experimental procedure is illustrated in Figure 1.

The ratings were made on a visual analog scale (VAS) programmed in MATLAB and displayed on the iPad. Two different VAS were displayed after each other. In the first, subjects were asked to answer the question “How pleasant was the smell?” This scale had the endpoints “not at all pleasant” and “very pleasant,” and was intended to measure the concept of “liking.” In the second VAS, subjects were asked to answer the question “How much do you want to smell this again?” This scale had the end points “not at all” and “very much,” and was intended to measure the concept of “wanting.” Each VAS scale disappeared as soon as the subject had given the rating, or otherwise after 5 s.

Prior to the experiment, at least 4 practice trials without exposition to odors were done so that the subjects got familiar with making the ratings on the iPad. In the main experiment, none of the participants exhibited problems with the ratings scales.

#### Questionnaires

Immediately after each experiment, subjects filled in two different questionnaires assessing several hedonic subjective features, administered in English. These questionnaires were the “BIS/BAS” Scale (Carver and White, 1994) and the “TEPS” (Gard, 2006).

The BIS/BAS Scale (“Behavioral Inhibition and Activation Systems” Scale) (Carver and White, 1994) is a 24-items questionnaire on a 4-point Likert scale (from 1 = “very true for me” to 4 = “very false for me”) that measures approach behavior (BAS) and avoidance/withdrawal (BIS). High BAS is generally associated with high positive affect in response to reward, while high BIS is associated with high negative affect in response to punishment (Gray and McNaughton, 1982).

The TEPS (“Temporal Experience of Pleasure Scale”) (Gard, 2006) is a measure specifically designed to capture the individual trait dispositions in both Anticipatory and Consummatory experiences of pleasure. Specifically, the Anticipatory scale is related to reward responsiveness and imagery, while the Consummatory scale is related to openness to different experiences, and appreciation of positive stimuli. It contains 18 statements about different hedonic situations that may occur in everyday life and it is measured on a 6-point Likert scale (from 1 = “very false for me” to 6 = “very true for me”).

For both questionnaires, participants were instructed that there were no right or wrong responses, but subjective ones related to each person's own experiences.

### Statistical analysis

All statistical analyses were made using SPSS Statistics version 21 (IBM, Chicago, USA). There were no specific hypotheses about how liking and wanting or sex may differentially affect the evaluation of the three odors. Therefore, the three odors were collapsed for the analyses.

#### Odor identification performance

The olfactory identification scores were compared between men and women using an independent samples *t*-test.

#### Analysis of change of ratings over time

First, it was investigated whether ratings could be predicted from the number of repetitions. This analysis was done separately for men and women. To this aim, linear regression analyses were performed on the single trial data with “liking” as the outcome variable and the number of repetitions per smell as the predictor. Subsequently, an analogous analysis was performed for the wanting ratings. For men, two piecewise linear regressions were performed separately for the ratings of the initial 4 trials and the remaining trials. This choice was made because the wanting ratings of men showed a steep decrease in the first trials before plateauing. For reasons of consistence, the same analysis was performed for the liking ratings.

#### Comparison of men and women

In order to determine whether the number of repetitions was a stronger predictor of the liking ratings for males than for females, a further linear regression analysis was performed with the single trials. In this analysis the regression coefficients of men and women were directly compared. We generated a dummy variable that was coded 1 for female and 0 for male (variable name “female”), one variable that contained the product of female and the liking ratings (variable name “femlike”), and a further variable that contained the product of female and the wanting ratings (variable name “femwant”). Then, “female,” “femlike,” and the liking ratings were used as predictors in the regression equation. In this way, the term “femlike” tests the null-hypothesis that the regression coefficients for females are the same as for men. The regression coefficients of men and women were compared twice, first between the women's slope for all trials and the men's slope for the first 4 trials (1–4), then between the women's slope for all trials and the men's slope for the remaining trials (5–40).

The same analysis was performed for wanting ratings, in order to compare the ratings between men and women. In this analysis, “female,” “femwant,” and the wanting ratings were used as predictors. Similar to the analysis of liking, the regression coefficients of men and women were compared twice, first between the women's slope for all trials and the men's slope for the first 4 trials (1–4), then between the women's slope for all trials and the men's slope for the remaining trials (5–40).

Whereas the regression analyses give information about the steepness of the change of ratings over time, they do not inform about the absolute rating values, i.e., whether an odor is rated as very pleasant or less pleasant in the beginning. To determine potential differences in these ratings, the second rating was selected for each subject and separately for wanting and liking ratings. The second rating was preferred to the first, because it was considered to be more reliable. The first rating may to a larger extent be influenced by novelty of the task. Also, the standard deviation of the very first rating appeared to be much higher than the standard deviation of the second rating. Moreover, despite the practice trials, subjects sometimes missed the first rating when the experiment started. Therefore, the second rating was used instead of the first.

The second rating was then submitted to a 2 × 2 repeated measures ANOVA with “evaluated aspect” (and the levels liking and wanting) as within-subjects factor and “sex” as between-subjects factor. Greenhouse-Geisser correction was used to adjust for violations of sphericity.

#### Questionnaires analyses

In order to determine whether there were any gender differences in the questionnaires results, a One-Way ANOVA between the scores of the questionnaires scales was computed. Level of significance was set to *p* < 0.05 for all analyses.

Spearman's correlations were computed separately for liking and wanting between the regression slopes, the second rating and the scores of the questionnaires scales. This procedure was done separately for men and women.

## Results

### Odor identification performance

Women had a higher sniffing sticks score than men, obtaining a mean sniffing sticks' score of 13.6 (*SD* = 1.4) and 12.6 (*SD* = 1.9), respectively. However, the difference between men and women was not significant (*t* = −1.27; *p* = 0.221).

### Analysis of change of ratings over time

On average, subjects missed answering 1.3 liking ratings and 1.9 wanting ratings within 120 trials.

**Figure 2:**
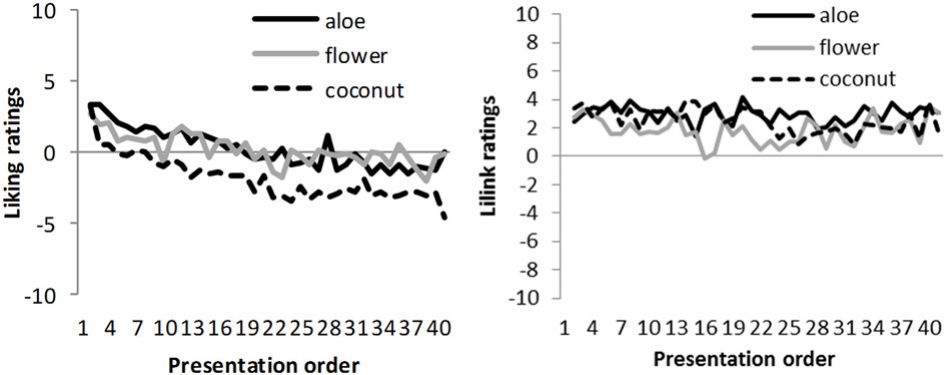
**Mean liking ratings of men (left) and women (right) across the number of repetitions**.

The *liking ratings* of women did not decrease with the number of repetitions (*t* = −1.52, *SE* = 0.01, *R* = 0.05, Beta = −0.05, *B* = −0.01, *p* = 0.128) (Figure 2). The liking ratings of men did not decrease significantly in trials 1–4 (*t* = −0.66, *SE* = 0.71, Beta = −0.11, *B* = −0.47, *p* = 0.516), but in the subsequent trials 5–40 (*t* = −6.43, *SE* = 0.01, Beta = −0.20, *B* = −0.04, *p* < 0.001). Thus, the liking ratings of men decreased significantly over repetitions from the 5th trial and could be predicted from the number of repetitions, whereas they maintained constant in women.

The *wanting ratings* of women decreased with the number of repetitions (*t* = −3.26, *SE* = 0.01, *R* = 0.10, Beta = −0.10, *B* = −0.02, *p* = 0.001). The wanting ratings of men decreased in trials 1–4 (*t* = −3.19, *SE* = 0.17, Beta = −0.30, *B* = −0.54, *p* = 0.002), but not in the trials 5–40 (*t* = −1.34, *SE* = 0.01, Beta = −0.04, *B* = −0.01, *p* = 0.182). Thus, after an initial steep decrease, wanting ratings maintained constant over repetitions.

### Comparison of men and women

Regarding *gender* differences, women's liking rating slopes did not significantly differ from that of men for trials 1–4 (*t* = −1.53, *SE* = 0.01, Beta = −0.05, *B* = −0.01, *p* = 0.127). Therefore, in the beginning of the experiment, liking ratings showed a similar pattern over repetitions for both sexes. However, liking ratings differed significantly between men and women in trials 5–40 (*t* = 4.08, *SE* = 0.01, Beta = 0.20, *B* = 0.03, *p* < 0.001). Thus, as the stimulation progressed, liking decreased more in men than in women, and the number of repetitions was a stronger predictor for liking in men than in women (Figure 2).

**Figure 3:**
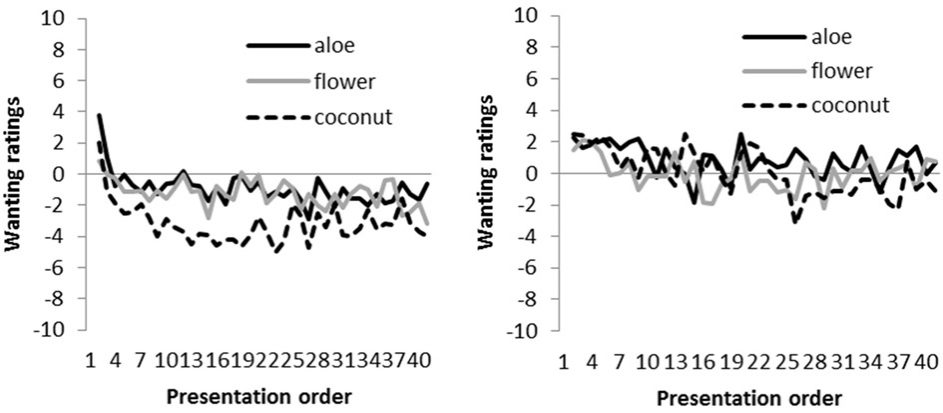
**Mean wanting ratings men (left) and women (right) across the number of repetitions**.

For the wanting ratings, men and women also showed different results, but in the opposite direction than for the liking ratings. The slope of women differed significantly from that of men for the trials 1–4 (*t* = 2.37, *SE* = 0.22, Beta = 2.60, *B* = 0.52, *p* = 0.018); decreasing at a steeper rate in men than women, but not for the trials 5–40 (*t* = −1.41, *SE* = 0.01, Beta = −0.08, *B* = −0.01, *p* = 0.159). Thus, after the first fast decrease in men, the wanting ratings showed a similar pattern over repetitions for both sexes (see Figure 3, for all trials).

The comparison of the second wanting and liking ratings showed a main effect with tendency toward significance of “evaluated aspects” [*F*_(1, 16)_ = 3.55; *p* = 0.078] but neither a significant main effect of “sex” [*F*_(1, 16)_ = 0.02; *p* = 0.881], nor a significant interaction between these two factors [*F*_(1, 16)_ = 0.22; *p* = 0.648]. This means that the second liking rating was slightly higher than the second wanting rating in both men and women (compare Table 2).

**Table 2:** **Mean values and standard deviations (*SD*) for questionnaire scales and ratings**.

	**Men**		**Women**	
	**Mean**	***SD***	**Mean**	***SD***
Liking ratings	4.76	2.39	6.22	1.98
Wanting ratings	4.10	2.06	5.14	2.53
BAS reward responsiveness	3.27	0.24	3.60	0.24
BIS	2.71	0.53	3.38	0.38
Initial liking ratings	6.28	2.00	6.69	1.67
Initial wanting ratings	5.66	1.91	6.24	2.17

### Questionnaires analyses

Significant sex differences were found for BAS Reward Responsiveness [One-Way ANOVA: *F*_(1, 16)_ = 8.33, *p* = 0.011] and BIS [*F*_(1, 16)_ = 9.45, *p* = 0.007] (compare Table 2). Women were found to be more sensitive to reward than men and also to be more oriented toward avoidance or withdrawal from negative stimuli.

No significant correlations were found between the second ratings and the slopes, neither for liking nor for wanting.

Correlations with the questionnaires scales, performed separately for men and women, showed significant correlations for both sexes (Table 3). In men, the slopes of the liking ratings 1–4 and BAS Reward Responsiveness (*r* = −0.68, *p* = 0.043) were significantly negatively correlated, as were the slopes 5–40 with BAS Reward Responsiveness (*r* = −0.72, *p* = 0.029), BIS (*r* = −0.71, *p* = 0.034) and TEPS Anticipatory scale (*r* = −0.82, *p* = 0.007). This means that the steeper the slope, the smaller men's reward responsiveness and reward anticipation. The slopes 1–4 of the wanting ratings were also negatively correlated with the BIS (*r* = −0.79, *p* = 0.012).

**Table 3:** **Correlations between ratings and questionnaires**.

	**Men (*N* = 9)**					
	**Second liking**	**Slope liking 1–4**	**Slope liking 5–40**	**Second wanting**	**Slope wanting 1–4**	**Slope wanting 5–40**
BAS drive	*r* = −0.01,	*r* = 0.15,	*r* = −0.14,	*r* = 0.23,	*r* = −0.07,	*r* = −0.01,
	*p* = 0.983	*p* = 0.695	*p* = 0.719	*p* = 0.560	*p* = 0.862	*p* = 0.974
BAS Fun Seeking	*r* = −0.08,	*r* = 0.47,	*r* = 0.02,	*r* = −0.09,	*r* = −0.15,	*r* = 0.51,
	*p* = 0.841	*p* = 0.199	*p* = 0.964	*p* = 0.822	*p* = 0.702	*p* = 0.161
BAS reward responsiveness	*r* = 0.34,	*r* = −0.68,	*r* = −0.72,	*r* = −0.09,	*r* = −0.62,	*r* = −0.32,
	*p* = 0.369	*p* = 0.043	*p* = 0.029	*p* = 0.814	*p* = 0.075	*p* = 0.400
BIS	*r* = 0.34,	*r* = −0.62,	*r* = −0.71,	*r* = −0.12,	*r* = −0.79,	*r* = −0.19,
	*p* = 0.370	*p* = 0.074	*p* = 0.034	*p* = 0.751	*p* = 0.012	*p* = 0.620
TEPS anticipatory	*r* = 0.22,	*r* = −0.41,	*r* = −0.82,	*r* = −0.01,	*r* = −0.41,	*r* = −0.27,
	*p* = 0.574	*p* = 0.273	*p* = 0.007	*p* = 0.974	*p* = 0.271	*p* = 0.491
TEPS consummatory	*r* = −0.23,	*r* = 0.47,	*r* = 0.11,	*r* = −0.26,	*r* = 0.45,	*r* = −0.02,
	*p* = 0.544	*p* = 0.203	*p* = 0.788	*p* = 0.498	*p* = 0.220	*p* = 0.966
	**Women (***N*** = **9**)**					
	**Second liking**		**Slope liking**	**Second wanting**		**Slope wanting**
BAS drive	*r* = 0.09,		*r* = 0.02,	*r* = −0.06,		*r* = −0.55,
	*p* = 0.811		*p* = 0.965	*p* = 0.879		*p* = 0.129
BAS fun seeking	*r* = 0.16,		*r* = −0.44,	*r* = −0.04,		*r* = −0.07,
	*p* = 0.690		*p* = 0.238	*p* = 0.930		*p* = 0.859
BAS reward responsiveness	*r* = −0.10,		*r* = 0.44,	*r* = −0.06,		*r* = −0.03,
	*p* = 0.793		*p* = 0.232	*p* = 0.878		*p* = 0.947
BIS	*r* = −0.15,		*r* = 0.26,	*r* = −0.04,		*r* = 0.41,
	*p* = 0.699		*p* = 0.507	*p* = 0.915		*p* = 0.279
TEPS anticipatory	*r* = 0.74,		*r* = −0.44,	*r* = 0.71,		*r* = −0.25,
	*p* = 0.023		*p* = 0.241	*p* = 0.031		*p* = 0.509
TEPS consummatory	*r* = 0.78,		*r* = −0.26,	*r* = 0.77,		*r* = −0.04,
	*p* = 0.014		*p* = 0.500	*p* = 0.016		*p* = 0.921

In women, no correlation between any slope and a questionnaire score was observed. However, women's second liking rating and the TEPS Anticipatory (*r* = 0.74, *p* = 0.023) and Consummatory (*r* = 0.78, *p* = 0.014) scales were significantly positively correlated. That means, the more women were reward-responsive, open to different experiences and appreciated positive stimuli, the higher their pleasantness ratings were at the beginning of the experiment. Moreover, in women, there were two significant positive correlations between the second wanting ratings and the TEPS Anticipatory (*r* = 0.71, *p* = 0.031) and Consummatory (*r* = 0.77, *p* = 0.016) scales: the more women were reward-responsive, open to different experiences and appreciating positive stimuli, the higher their wanting ratings were at the beginning.

## Discussion

It has been suggested that “liking,” the actual affective or hedonic experience, differs from “wanting,” the motivation or urge to make such experiences (Berridge and Robinson, 2003). The present study aimed to determine whether such a difference could also be observed for olfactory stimuli, i.e., pleasant odors. In addition, we were interested in whether there are potential gender differences in the appreciation of pleasant odors over repetitions.

Our results suggest that wanting and liking are different concepts also in the domain of olfaction. Firstly, at the first contact with the olfactory stimulus, the degree of pleasantness is evaluated slightly higher than the willingness to be exposed further to it. More importantly, liking and wanting changed differently over time. Women “liked,” i.e., continued to find the odors pleasant during the entire experiment, although they did not wish (“want”) to smell the odors again after a while. Thus, liking persisted in women even after 120 odor presentations in total (40 repetitions per odor), but wanting decreased.

Differently to women, men's liking decreased only slightly during the first 4 expositions, but more steeply afterwards. This fast decrease in liking after the first 4 trials in men was related to both reward responsiveness and reward anticipation. The steeper the slope with which ratings decreased, the less the individual was responsive to reward. Wanting, in contrast, decreased steeply during the first 4 expositions, but to a much lesser extent for the remaining trials. Thus for men, the very initial ratings are enough in order not to want being exposed to the same olfactory stimulation again, while afterwards the ratings are still maintained low but constant. Altogether, wanting and liking developed differently across repetitions in both men and women.

### Differences between men and women

In addition to these differences between wanting and liking that were observed in both men and women, there were also sex differences for liking and wanting. Women showed a smaller decrease in liking over repetitions than men. This may be due to the fact that smells are more important for women than for men (Croy et al., 2010), or to the fact that women are more interested in odors than men (Seo et al., 2011). This may be related to the finding that women's second liking and wanting ratings were significantly correlated to anticipatory and consummatory experiences of pleasure in the current study. We speculate that, when approaching pleasant odors, the anticipatory pleasure of those women who are more reward sensitive may have led to high expectations, thus, high initial liking and wanting ratings, and to their constant maintenance over repetitions. However, replications with a larger number of subjects would be required.

It has also been suggested that women are more attentive to odors than men from an early age (Ferdenzi et al., 2008), and that women evaluate odors as more important than men, for example when selecting a potential partner (Herz and Inzlicht, 2002). Moreover, hormonal factors associated with gender differences may also modulate the perceived pleasantness of odors (Rouby et al., 2009).

Women and men also differed regarding wanting. Wanting is conceptualized as the consequence of a process that assigns value to perceptual events (Berridge and Robinson, 2003). During this process, sensory and cognitive information is transformed into attractive and desirable entities. The fact that liking changed in a different way for men and women may indicate that the repeated stimulation leads to different hedonic experiences in women and men, but only after relatively long periods of time. Indeed, at the very beginning of the stimulation, both men and women liked the odors to the same degree, whereas after a short while only the men did not like the odors anymore. On the contrary, wanting was processed differently only at the beginning of the stimulation, with men showing a sort of “instantaneous rejection” which settled down afterwards, while women showed a more constant decrease. Thus, after the initial ratings, men and women behaved in the same way: they did not wish to smell the odors again and this feeling maintained constant until the end of the experiment. These results support the idea that liking and wanting are two related but different concepts which also act differently between sexes. In an applied context, women may continue to experience the repeated exposure to a perfume in odorized shop as pleasant. In the same way, the repeated smell of a perfume may decrease the wish to buy it, and this may take much less time in men than women, both because men start experiencing the smell as less and less pleasant already after the very first expositions and because they would avoid to smell it again already after a short while.

In addition to the small sample size, the present study is limited by the lack of an intensity measure which makes it difficult to estimate the influence of habituation. However, we assume that habituation was unlikely to induce the observed variation of pleasantness, since we had an ISI of 18 s, and long ISIs have previously been found to prevent habituation (Croy et al., 2013a). Moreover, the different odors were always presented alternatingly, thereby further disrupting a possible process of habituation. Moreover, habituation does not imply that perception disappears. If an odor would not be detectable (perceivable) anymore, its rated pleasantness should be at around 0, which is the neutral baseline. This was not the case in the present study, which suggests that the change in pleasantness ratings cannot solely be attributed to a decrease in detectability.

Finally, the constant order of the “liking” and “wanting” scales may have induced effects of the first on the second evaluation. Nevertheless, the ratings for liking and wanting were significantly different. Thus, even though we cannot exclude an influence of the liking ratings on the wanting ratings, we still found evidence for the two concepts being different, even in the domain of olfaction.

Summing up, the experienced pleasantness for olfactory stimuli showed a steeper decrease over repetitions for men than women. Further studies should investigate whether liking and wanting also differ in other sensory modalities than taste and smell, and possibly also between men and women.

### Conflict of interest statement

The authors declare that the research was conducted in the absence of any commercial or financial relationships that could be construed as a potential conflict of interest.
